# Arbuscular mycorrhiza fungi increased the susceptibility of *Astragalus adsurgens* to powdery mildew caused by *Erysiphe pisi*

**DOI:** 10.1080/21501203.2018.1477849

**Published:** 2018-05-28

**Authors:** Yuanzheng Liu, Xi Feng, Ping Gao, Yanzhong Li, Michael J. Christensen, Tingyu Duan

**Affiliations:** State Key Laboratory of Grassland Agro-Ecosystems, Lanzhou University, Lanzhou, China; National Demonstration Center for Experimental Grassland Science Education, Lanzhou University, Lanzhou, China; College of Pastoral Agriculture Science and Technology, Lanzhou University, Lanzhou, China

**Keywords:** Standing milkvetch, Arbuscular mycorrhizal fungi, Powdery mildew, biocontrol

## Abstract

Powdery mildew caused by *Erysiphe pisi* is a major factor that affects the growth of standing milkvetch (*Astragalus adsurgens*). As arbuscular mycorrhizal fungi (AMF) have shown to be enhancing the resistance of plants to biotrophic pathogens such as powdery mildew, a study was carried out to look at the effects of three AMF, either singularly or in combination, on the growth of standing milkvetch and susceptibility to *E. pisi*. The results showed that the presence of AMF enhanced the growth of standing milkvetch even though their presence in the roots increased susceptibility to this foliage pathogen compared with plants having no AMF. This increase in growth of plants with severe infection of powdery mildew was especially surprising as leaves contained lower levels of chlorophyll than plants without AMF and had a greater concentration of malondialdehyde, an indicator of the damage of cell membrane. The effects on the extent of growth and powdery mildew enhancement differed inconsistently with the type of AMF in roots. The effects on growth and powdery mildew were not related to intensity of AMF colonisation. The peroxidase (POD) was consistently higher activity (15% to 72%) in plants with AMF than plants without them.

## Introduction

Standing milkvetch (*Astragalus adsurgens*) is an important perennial legume, which is widely distributed in the USA, Canada, Russia, China, Mongolia, Korea and Japan (Gao et al. 2001; Liu et al. 2015). In China, it has been widely cultivated in arid, semi-arid, sandy and desert areas, especially in the Loess Plateau area since the 1970s (Hou and Bai 1986; Su 1985). Standing milkvetch is used as forage because of its high nutritional value and abundant protein content. Moreover, it is used for windbreaks to prevent the movement of sand and protect the soil from erosion. It also serves as green manure and a nectar-producing plant (Li and Nan 2007, 2008).

Powdery mildews are the most frequently encountered plant pathogenic fungi worldwide (Braun 1995; Zheng and Yu 1987), infecting the aboveground parts of more than 10,000 plant species (Glawe 2008) including many legume crops such as standing milkvetch (Nan and Li 1994). Powdery mildew of standing milkvetch is caused by the ascomycete *E. pisi* (Zheng and Yu 1987). In a study of pathogen effects on standing milkvetch, *E. pisi* decreased plant biomass by approximately 30% to 70%, and plant crude protein by 11%, and increased plant coarse fibre content by 12.9% (Nan and Li 2003). This pathogen is now recognised as a major factor adversely affecting the growth of standing milkvetch in China (Duan et al. 2015).

Arbuscular mycorrhizas (AM) occur widely in terrestrial plant species, including legume species. The symbiosis is normally mutualistic, based primarily on enhanced plant mineral nutrient uptake, particularly phosphorus (Smith and Read 2008); they can also improve resistance to disease (Newsham et al. 1995), especially soil-borne diseases such as those caused by *Fusarium oxysporum* (Wang et al. 2012), *Rhizoctonia solani* (Huang et al. 2006) and *Phytophthora parasitica* (Pozo et al. 2002; Vigo et al. 2000). Studies on the interactions of AMF and aboveground diseases have also shown that AMF can reduce the severity of powdery mildew disease. An example of this occurred in pea (*Pisum sativum*), where inoculation with *Glomus mosseae* decreased the incidence of powdery mildew (*E. pisi*), and reduced the disease index (DI) from 55.2% to 28.7% (Singh et al. 2004). In another study, inoculation of wheat (*Triticum stivum*) with *Funneliformis mosseae* and *Rhizophagus irregularis* reduced the number of *Blumeria graminis* f. sp. *tritici* conidia with haustoria, and resulted in accumulation of polyphenolic compounds at infection sites of *Blumeria graminis* f. sp. *tritici*, reducing the occurrence of powdery mildew. Protection imparted by the two AMF species reached 74% and 34%, respectively, compared with the control (Mustafa et al. 2016). Similar results were found in apple tree seedlings, where soil inoculation with a mixture of AMF species decreased powdery mildew (*Podosphaera leucotricha*) infection to a similar extent to the fungicide Flint (trifloxystrobin), and better than the strobilurin fungicide Strobyon (Yousefi et al. 2011). Possible defence mechanisms induced by AMF include increased plant nutrient acquisition, damage compensation, competition with pathogens for carbohydrates and plant photosynthates, induction of plant biochemicals such as phenolics and expression of disease-resistance genes (Feldmann and Boyle 1998; Ruiz-Lozano et al. 1999). AMF-induced changes in the expression of plant disease–related enzymes, such as peroxidase (POD), catalase (CAT) and polyphenol oxidase (PPO), and decreases in the levels of malondialdehyde (MDA), an indicator of damage to membranes from lipid peroxidation, have been reported in perennial ryegrass (*Lolium perenne*) infected with the foliage pathogen *Bipolaris sorokiniana* (Li et al. 2018). Both POD and CAT are plant-protection enzymes, which reduce reactive oxygen species and can alleviate damage sustained by plants exposed to some biotic and abiotic stresses (Li et al. 2018). MDA is a marker for membrane lipid peroxidation, and is correlated with plant disease resistance (Liu et al. 2014).

Although powdery mildew has been shown in trials to be extremely damaging to standing milkvetch in the field, this species is nevertheless a productive and persistent forage plant and helps to stabilise the region where it grows. This study was conducted to investigate if AMF in the roots of standing milkvetch reduces the severity of powdery mildew. The effects of three AMF commonly used in studies, *Claroideoglomus etunicatum, G. versiforme* and *F. mosseae*, individually, dual-mixed or triple-mixed, against powdery mildew caused by *E. pisi* on standing milkvetch were assessed. We hypothesised that (1) AMF will improve standing milkvetch growth, (2) AMF will decrease the occurrence of powdery mildew on standing milkvetch and that the three AMF inoculated individually and together will confer different levels of protection against this powdery mildew disease.

## Materials and methods

### Plants and fungi

The species used was standing milkvetch (*Astragalus adsurgens* var. *Shanxi Yulin*). The AMF were *C. etunicatum, G. versiforme* and *F. mosseae*, which were obtained from the Institute of Plant and Environmental Resources of the Chinese Academy of Agriculture, Beijing China. Inoculum was prepared from pot cultures of the AMF grown on *Trifolium subterraneum* in the same soil mix as used for the experiments, and consisted of dry soil containing spores and colonised root fragments.

### Experimental design

Standing milkvetch individuals were grown as (1) single-inoculated plants with one of the three AMF, (2) plants inoculated with dual-mixed AMF, (3) plants inoculated all three AMF mixed together (triple-mixed inoculation) and (4) non-inoculated (NM). In total, there were seven AMF treatments and one NM treatment, consisting of eight pots for each inoculation treatment, with a total of 64 pots. For the AMF inoculation treatments, a total of 21.0 g of single inoculum (or 10.5 g of each AMF for dual-mixed inoculation or 7.0 g of each AMF for the triple-mixed inoculation) was mixed with the growth medium. The NM treatment had 21.0 g of sterilised AMF inoculum. The pot size for all treatments was 20-cm tall, 12-cm wide at the bottom and 15-cm wide at the top. Each pot contained 1.5 kg of growth medium.

### Growth medium

Soil collected from the Xinlong Mountain, Lanzhou, China, was used to prepare the soil mix used in the experiments. The mix consisted of a mixture of 10% soil and 90% fine sand. All components were sieved through a 2-mm sieve, and the soil and sand mix was autoclaved at 121℃ for 1 h twice over a period of 3 days and then dried in an oven at 180 ℃ for 36 h. The mix (hereafter referred to as soil) had 6.6 mg P kg^−1^ plant-available P, 120 mg kg^−1^available N, 40 mg kg^−1^ K and a pH of 6.2.

### Plant growth and harvesting

The seeds of standing milkvetch were first sterilised in 75% (v/v) ethanol for 1 min, then soaked in 3% (v/v) NaClO for 5 min and washed five times with sterilised water. The sterilised seeds were placed on wet filter paper and incubated in the dark at 20℃ for 48 h to germinate. Six germinated seeds were planted in each pot, and thinned to three seedlings per pot after 2 weeks. Plants were then left to grow and naturally become infected with powdery mildew. The leaves were counted and plant height was measured every week to determine the growth of plants in each treatment.

We conducted the experiment in a glasshouse with irradiance in the range of 180–850 mmol m^−2^ s^−1^, from April to July. The temperatures were 23–28 °C (day) and 20–25 °C (night). Plants were watered every day to maintain 10% (w/v) soil water content for each pot. We applied a modified Long Ashton nutrient solution (-P; Duan et al. 2011) to the pots every other day during the experiment. The plants were grown for 12 weeks.

### Disease assessment

Powdery mildew was surveyed before the harvest. Ten branches from the three plants in each pot were randomly selected and all the leaves on the 10 branches were used to assess the disease incidence and severity. Powdery mildew severity was recorded as four discrete levels by visually estimating the percentage of observed leaves covered by pustules: 0 for no signs of infection; 1 for 0.1% to 5% of leaf area covered with mycelium; 2 for 5.1% to 20%; 3 for 20.1% to 50%. DI was calculated using the formula:

$$DI\;=\;100\;\;\times \sum_{n=i}^{i}(n\;\times Ln)\;/\;5\times LN$$

where *i* is the scale of disease severity (*i* = 0, 1, 2, 3, 4), *LN* is the total number of leaves and *Ln* is the number of leaves in each disease severity ranking.

### Enzyme analyses

The plants were then harvested by cutting at ground level. Shoots from the three plants of each pot were combined, and the fresh weight (FW) was determined. Two subsamples of the combined branches of each pot were taken and each was weighed. One of the samples was used for enzyme and MDA analyses, and the other was oven dried to obtain the dry weight, which was used to estimate the total dry weight of the plants in each pot. The activity of POD and PPO, and the MDA content, was determined using an ultraviolet spectrophotometer (Pectrum, Shanghai 721, China). POD activity and chlorophyll content was determined at 470 nm and 652 nm, respectively (Li et al. 2000). We determined PPO activity at 495 nm using the method described in Kumar and Khan (1982). MDA was measured at 600 nm, 450 nm and 532 nm using the method described by Li et al. (2000). CAT activity was measured using another UV spectrophotometer (Perkin Elmer-Lambda 25-USA) at 240 nm following the published method (Beers and Sizer 1952).

Roots were carefully washed, blotted and the fresh weights were determined. One of the weighed subsamples was taken to determine the total root dry weight (from the fresh weight: dry weight ratios from the subsamples). The weighed subsample (~0.1 g) was cleaned in 10% (w/v) KOH, stained in 1% (w/v) trypan blue (Phillips and Hayman 1970) and used to assess AMF colonisation using the grid-intersect method described by Giovannetti and Mosse (1980).

### Statistical analysis

Data are presented as means and standard errors of means of eight replicates. Data were analysed by analysis of variance (ANOVA) using SPSS 19.0 statistical analysis software (SPSS Inc., Chicago, USA). Comparisons between means were based on the least significant differences at the 0.05 probability level. Data for percent AM colonisation were ARCSIN-transformed to achieve normality.

## Results

### Effects of AMF on plant growth

**Figure 1.**
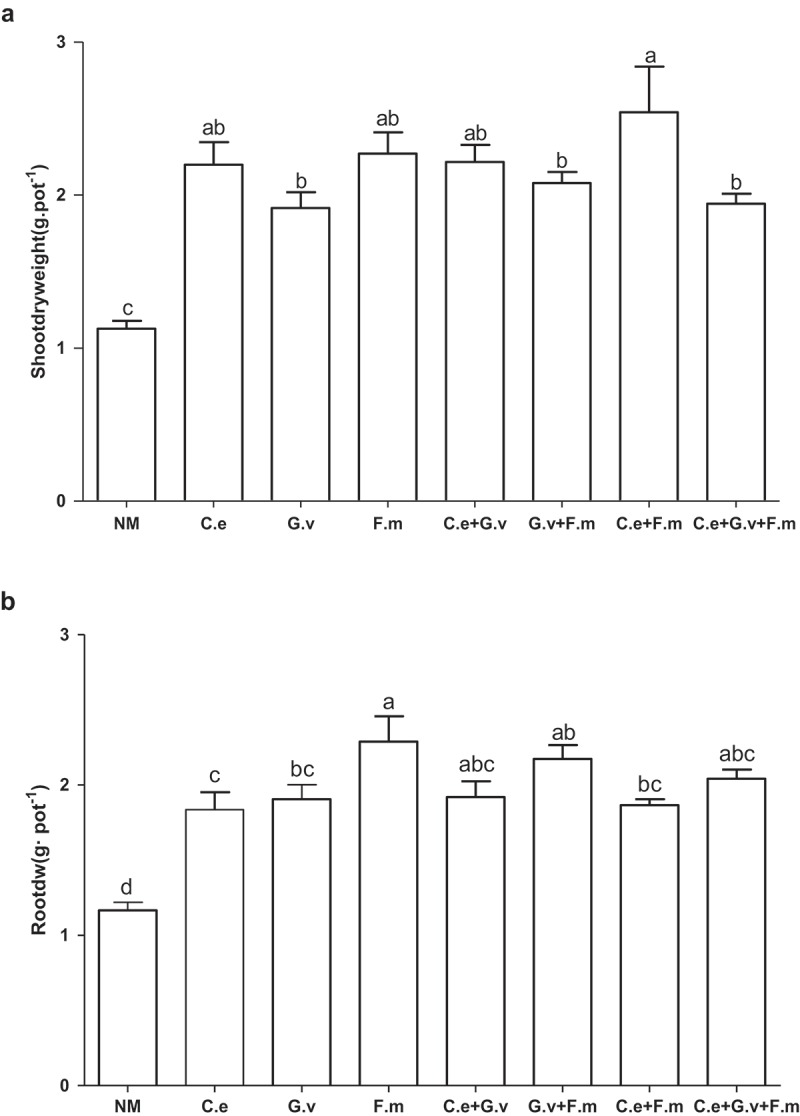
Shoot biomass (a) and root biomass (b) of standing milkvetch (*Astragalus adsurgens*) infected by *Erysiphe pisi*, colonised by arbuscular mycorrhizal fungi (AMF) *Claroideoglomus etunicatum* (*C. e), G. versiforme* (*G. v), Funnelliformis mosseae* (*F. m*) and the mixes of three AMF, NM = not inoculated with AMF. Bars topped by the same letter do not differ significantly at *p *< 0.05 by ANOVA test.

On average, the inoculation of AMF increased shoot dry weight, root dry weight and total dry weight of plants by greater than 55% relative to AMF-free controls (Figure 1; Supplementary Figure 1, Supplementary Table)(*p *< 0.05). Inoculation with all AMF treatments increased the aboveground biomass of standing milkvetch between 69.9% to 125% (*p *< 0.05) depending on the AMF treatment applied. Of all the inoculated AMF treatments, plants inoculated with a mixture of *F. mosseae* + *G. versiforme* had the highest shoot biomass, significantly higher than that of *G. versiforme, G. versiforme* + *F. mosseae*, and the triple-mixed inoculation (*p *< 0.05; Figure 1(a)) where shoot biomass increased by 16% to 33%.

Inoculation with AMF also significantly increased plant root dry weight from 50% to 110%, with plants inoculated with *F. mosseae* having the highest root dry weight and this increase was on average 20% higher than that of plants inoculated with *C. etunicatum, G. versiforme* and the mix of *G. versiforme* + *F. mosseae* (*p *< 0.05). The other AMF treatments had similar increases in root dry weight (Figure 1(b)). Compared with the NM, only the treatment with the mix of *F. mosseae* and *C. etunicatum* significantly increased the root-to-shoot ratio (Supplementary Figure 1).

### AM fungal colonisation

**Figure 2.**
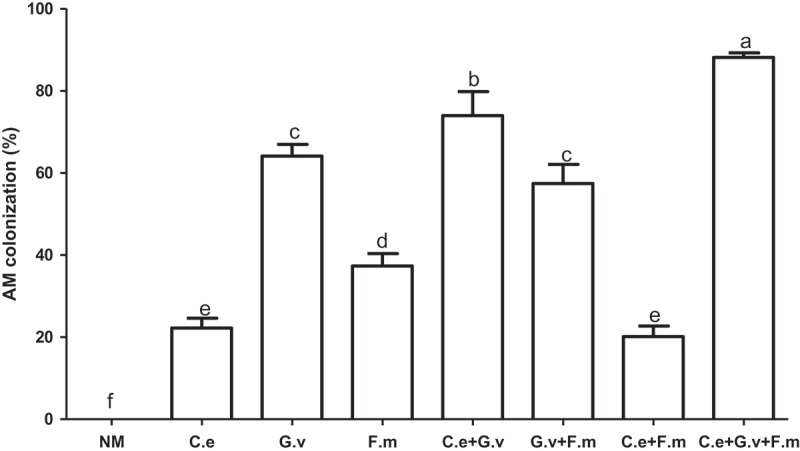
AM colonisation of standing milkvetch (*Astragalus adsurgens*) infected by *Erysiphe pisi*, colonised by arbuscular mycorrhizal fungi (AMF) *Claroideoglomus etunicatum* (*C. e), G. versiforme* (*G. v), Funnelliformis mosseae* (*F. m*) and the mixes of three AMF, NM = not inoculated with AMF. Bars topped by the same letter do not differ significantly at *p *< 0.05 by ANOVA test.

All inoculated standing milkvetch plants in the experiment were colonised after AMF inoculation and NM controls remained free of mycorrhizal fungi for the duration of the experiment (Figure 2). AMF colonisation of roots inoculated with the triple AMF mix was significantly higher than other treatments (*p *< 0.05), ranging from 0.24 to 4.3 fold higher than NM controls. The next highest coloniser was *G. versiforme*, followed by the dual mixes of *G. versiforme* with either *F. mosseae* or *C. etunicatum. C. setunicatum* alone, and the mix of *C. etunicatum + F. mosseae* had the lowest levels of AM colonisation (*p *< 0.05) (Figure 2).

### Powdery mildew occurrence in relation to AMF colonisation

**Figure 3.**
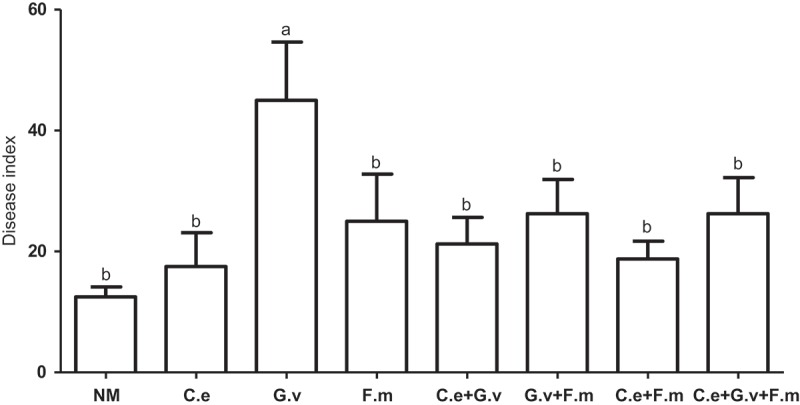
Disease index (DI) of powdery mildew (*Erysiphe pisi*) of standing milkvetch (*Astragalus adsurgens*), colonised by arbuscular mycorrhizal fungi (AMF) *Claroideoglomus etunicatum* (*C. e), G. versiforme* (*G. v), Funnelliformis mosseae* (*F. m*) and the mixes of three AMF, NM = not inoculated with AMF. Bars topped by the same letter do not differ significantly at *p *< 0.05 by ANOVA test.

Obvious symptoms of powdery mildew occurred first in the eighth week after plant emergence; the disease progressed slowly and white colony areas on leaves were less than 50% of the total leaf areas at harvest. The pathogen was identified as *E. pisi*, based on the morphology as described by Zheng and Yu (1987). In general, inoculation with AMF tended to increased the DI of standing milkvetch powdery mildew, however, only *G. versiforme* root colonisation increased powdery mildew disease significantly above the NM control (*p *< 0.05) (Figure 3).

### Effects of AMF on POD, CAT and PPO activity

**Figure 4.**
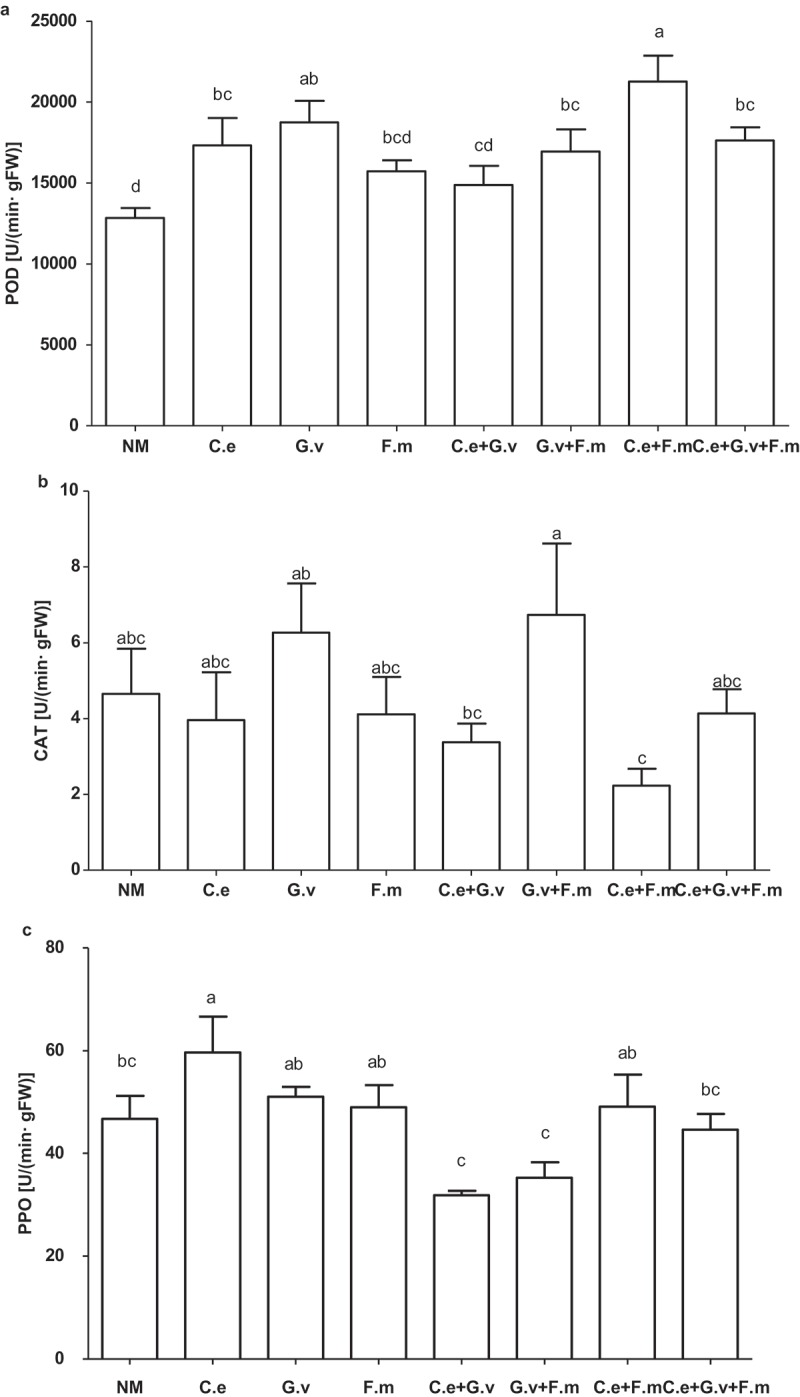
Peroxidase (POD) (a), catalase (CAT) (b) and polyphenol oxidase (PPO) (c) of standing milkvetch (*Astragalus adsurgens*) infected by *Erysiphe pisi*, colonised by arbuscular mycorrhizal fungi (AMF) *Claroideoglomus etunicatum* (*C. e), G. versiforme* (*G. v), Funnelliformis mosseae* (*F. m*) and the mixes of three AMF, NM = not inoculatedwith AMF. Bars topped by the same letter do not differ significantly at *p *< 0.05 by ANOVA test.

AMF significantly affected the activity of the studied enzymes (Figure 4, Supplementary Table) in standing milkvetch leaves. Compared with the NM treatment, inoculation with AMF significantly increased the activity of POD in leaves except for plants inoculated with *F. mosseae*, and *C. etunicatum + G. versiforme* (*p *< 0.05). Of the AMF treatments, plants inoculated with *C. etunicatum*+ *F. mosseae* and *C. etunicatum*+ *G. versiforme* had the highest and lowest POD activity, being 72% and 15% higher than that NM treatment, respectively (*p *< 0.05; Figure 4).

Compared with the NM, AMF treatments significantly altered CAT activity in leaves. Plants inoculated with *G. versiforme* + *F. mosseae* had the highest CAT activity, being 0.2-to-3-fold greater than other treatments. Compared with the NM, only *C. etunicatum* colonisation induced PPO activity (*p *< 0.05).

### MDA and chlorophyll content

**Figure 5.**
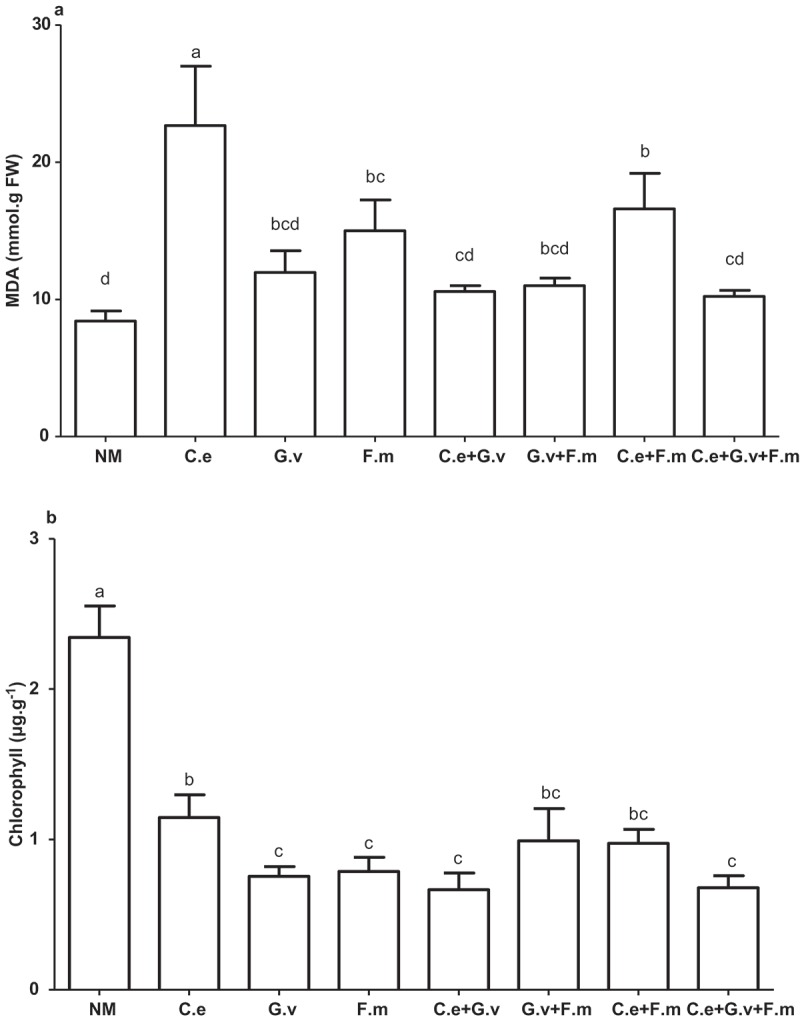
Malondialdehyde (MDA) (a) and Chlorophyll (Chll) (b) of standing milkvetch (*Astragalus adsurgens*) infected by *Erysiphe pisi*, colonised by arbuscular mycorrhizal fungi (AMF) *Claroideoglomus etunicatum* (*C. e), G. versiforme* (*G. v), Funnelliformis mosseae* (*F. m*) and the mixes of three AMF, NM = not inoculated with AMF. Bars topped by the same letter do not differ significantly at *p *< 0.05 by ANOVA test.

Compared with the NM, AMF treatments *C. etunicatum, F. mosseae*, and *C. etunicatum + F. mosseae* significantly increased MDA concentrations in shoots (*p *< 0.05), most notably in the *C. etunicatum* treatment, which had the highest MDA concentration. The MDA concentrations in shoots of the other AMF treatments, although trending higher, were not significantly different from the plants of the NM treatment at the 5% level (Figure 5(a)). The NM had the highest plant leaf chlorophyll concentration, and was 51.12–71.62% higher than that of the AMF treatments (*p *< 0.05), with the next highest concentration in plants inoculated with *C. etunicatum* alone. The other AMF treatments had similar but trending lower chlorophyll concentrations (Figure 5(b)).

## Discussion

The present study revealed that the inoculation of AMF individually, dual-mixed or triple-mixed enhanced plant growth while, at the same time increased the susceptibility of standing milkvetch to *E. pisi*. This increase occurred irrespective of the species or combinations of AMF inoculated. The increase in growth of plants inoculated with AMF is not surprising as this positive effect on plants hosting AMF has been commonly reported (Klironomos 2003). However, to have increased growth when the presence of AMF makes the host plants more badly affected by a fungal pathogen was not expected.

The failure of AMF to decrease the occurrence of powdery mildew on standing milkvetch contrasts with some previous studies on the effects of AMF on the severity of powdery mildew in host plants. The few studies which directly investigated the effects of AMF against powdery mildew in plants showed that inoculation with AMF either reduced the intensity of powdery mildew disease or had no effect on disease intensity. Specifically, Singh and colleagues (2004) found that *F. mosseae* reduced the severity of powdery mildew on peas, and research conducted by Mustafa and colleagues (2016) showed that *F. mosseae* and *R. irregularis* reduced the severity of powdery mildew on wheat. Another study showed that the impact of the AMF plant symbiosis on powdery mildew infection of cucumber (*Cucumis sativus*) varied from no effect to reduction of infection of the foliage pathogen (Chandanie et al. 2006).

The increase in growth of plants with AMF, even though in all treatments the presence of these fungi in the roots resulted in greater severity of powdery mildew disease than plants without AMF, is even more surprising when one looks at the levels of chlorophyll and MDA in the leaves. In leaves of plants of all AMF treatments, the levels of chlorophyll were lower than that of the non-AMF control plants. Thus, it seems logical that lower chlorophyll levels would result in reduced photosynthesis and lower production of usable carbohydrate, consequently. Hence, the plants would have reduced growth. And in leaves in plants of all of the AMF treatments, the content of MDA was higher than of the no-AM fungal control plants. This indicates that there was more cell membrane damage in plants with AMF than in the no-AM fungal plants. This increased damage of cell membrane would also seem likely to result in reduced plant growth.

Both *E. pisi* and the AMF are obligate biotrophs and so obtain all of their required nutrients from the host plant. Presumably, this comes at a cost to the host plants, but with AMF their presence is typically of benefit; particularly, when low levels of available phosphate are available in the soil. Infection of standing milkvetch by powdery mildew is a real cost to the plant as previously mentioned, its presence has been found to cause losses in plant yield of between 30 and 70% (Nan and Li 2003). However, this present study found that the presence of AMF in the roots mitigates the yield loss caused by the powdery mildew infecting the leaves. And thus, although it would appear from the disease values that the AMF were not beneficial to standing milkvetch, their presence is of real benefit for the alleviation of powdery mildew disease effects based on the growth of both the foliage and roots.

Another important finding of this study was that AMF’s effects on plant growth and pathogen susceptibility appeared to be independent of the AMF species makeup, either singularly or in combination.

Our research also showed that the intensity of AM fungal colonisation was not consistently related to plant shoot or root dry weight. For example, plants inoculated with *C. etunicatum* + *F. mosseae*, and with *F. mosseae*, had the lowest and the second lowest AM colonisation, respectively, but had the highest values of shoot and root dry weight. This contrasts with a previous study, which found that the extent of AM fungal colonisation was related to the occurrence of powdery mildew and showed a negative correlation; a low degree of AMF root colonisation prior to inoculation with *Oidium sp*. was correlated with a high susceptibility of plants to powdery mildew; whereas, a lower degree of pathogen infection was associated with a high AM fungal colonisation (Feldmann and Boyle 1998).

Examination of the activity of three enzymes linked to disease resistance of host plants has failed to find any connection with the presence of AMF and the intensity of powdery mildew disease. This study shows that AMF did not consistently increase the activity of CAT and PPO relative to that of the plants without AMF. Only with POD did plants of all AMF treatments have higher activity than the no AMF control plants. Thus, in this regard, standing milkvetch responded differently to other plants colonised by AMF (Maya and Matsubara 2013).

## Conclusions

This study showed that the presence of AMF consistently increased the shoot and root growth of standing milkvetch even though their presence in the roots increased susceptibility to powdery mildew. This increase in growth was thus surprising especially as plants with AMF and high powdery mildew infection has lower chlorophyll content and higher MDA content than plants with AMF. Field growing standing milkvetch plants will host AMF in their roots; in addition, their presence may explain why it is a persistent and productive forage plant even though a greenhouse-based study found that powdery mildew disease severely reduced its growth. Research is required to understand how the presence of AMF can provide such benefits to plant growth even though plants became more severely infected with this foliage pathogen.
